# Supplementary material for: Mate selection: A useful approach to maximize genetic gain and control inbreeding in genomic and conventional oil palm (Elaeis guineensis Jacq.) hybrid breeding
Source: PLoS Comput Biol. 2023 Sep 11;19(9):e1010290. doi: 10.1371/journal.pcbi.1010290 (PMC10513302; doi:10.1371/journal.pcbi.1010290)
Supplement: S1 Fig — (DOCX) [file pcbi.1010290.s001.docx]

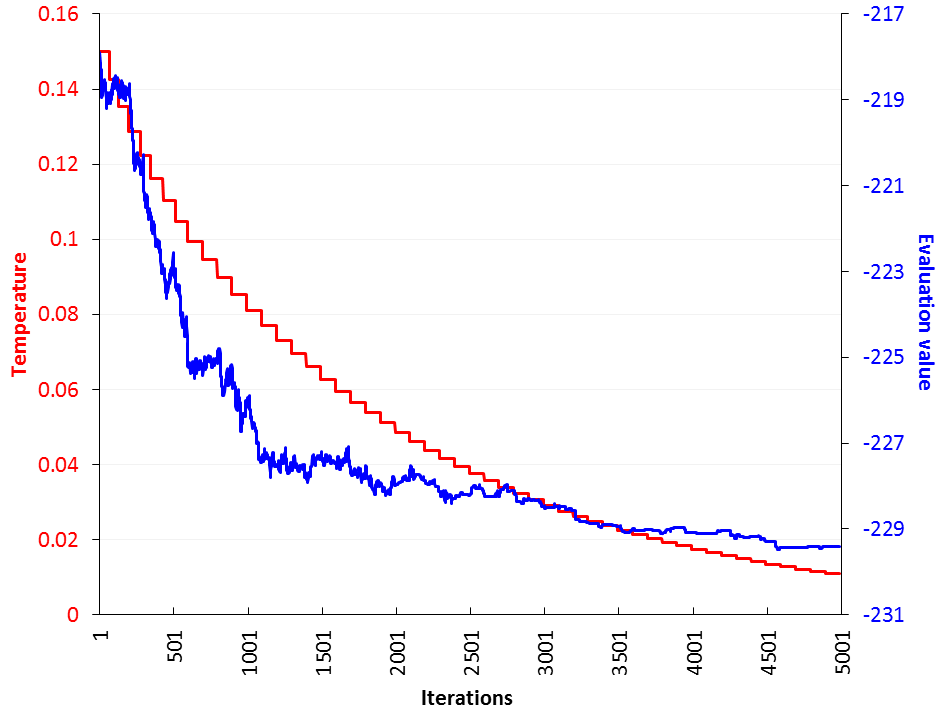


S1 Fig Example result of evolution of evaluation values and temperature according to iterations, until convergence
