# Supplementary material for: Mate selection: A useful approach to maximize genetic gain and control inbreeding in genomic and conventional oil palm (Elaeis guineensis Jacq.) hybrid breeding
Source: PLoS Comput Biol. 2023 Sep 11;19(9):e1010290. doi: 10.1371/journal.pcbi.1010290 (PMC10513302; doi:10.1371/journal.pcbi.1010290)
Supplement: S3 Fig — Figures are means over 30 replicates. (DOCX) [file pcbi.1010290.s003.docx]

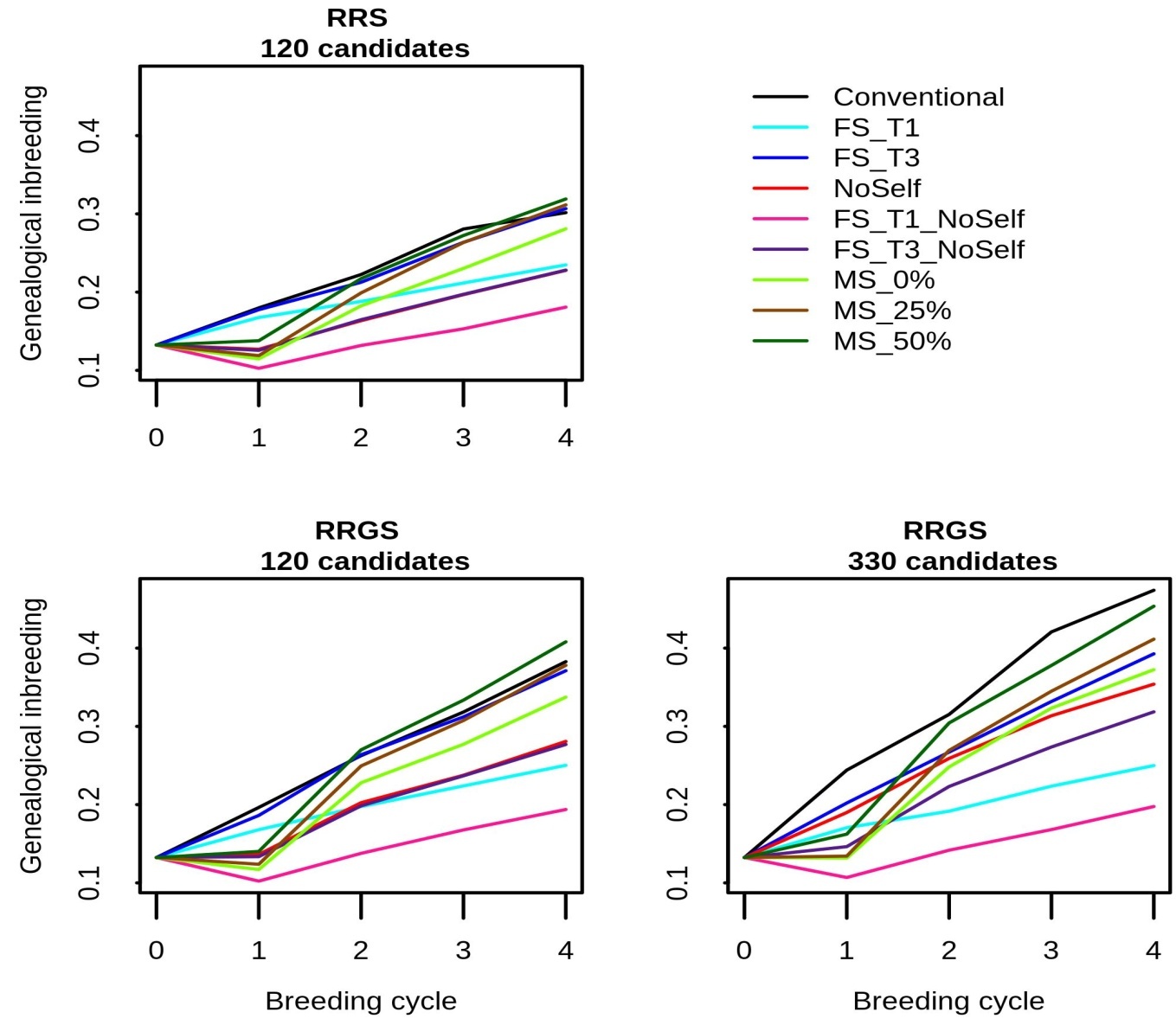


S3 Fig Genealogical inbreeding in the La Mé population according to the generations (0-4), breeding methods (RRS with 120 candidates, RRGS with 120 candidates and RRGS with 330 candidates) and methods of selection and mating. Figures are means over 30 replicates.
