# Supplementary material for: Mate selection: A useful approach to maximize genetic gain and control inbreeding in genomic and conventional oil palm (Elaeis guineensis Jacq.) hybrid breeding
Source: PLoS Comput Biol. 2023 Sep 11;19(9):e1010290. doi: 10.1371/journal.pcbi.1010290 (PMC10513302; doi:10.1371/journal.pcbi.1010290)
Supplement: S4 Fig — Figures are means over 30 replicates. Values with the same letters are not significantly different within a breeding method at P = 5%. (DOCX) [file pcbi.1010290.s004.docx]

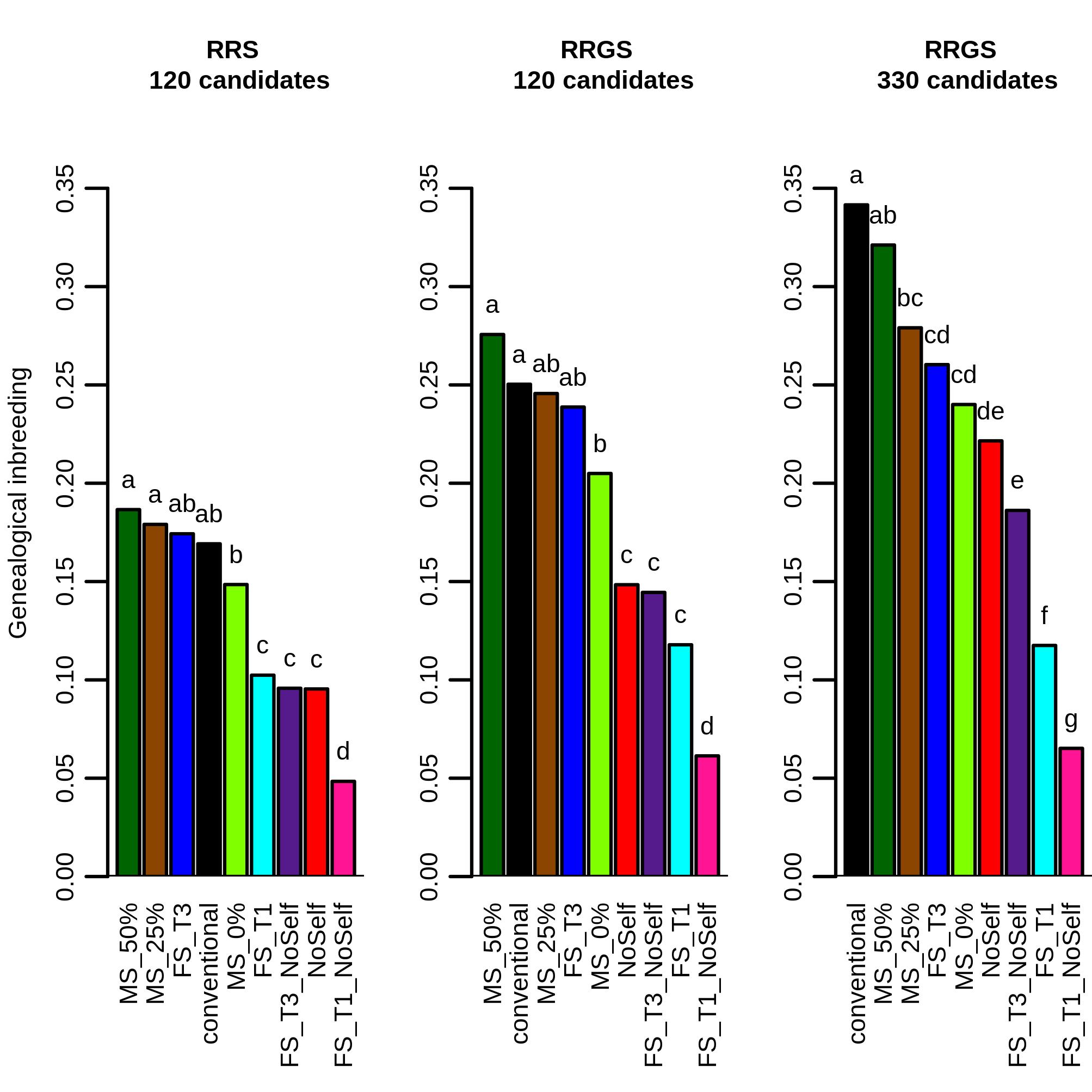


S4 Fig Genealogical inbreeding in the La Mé population after four breeding cycles according to method of selection and mating and breeding scheme (RRS with 120 candidates, RRGS with 120 candidates and RRGS with 330 candidates). Figures are means over 30 replicates. Values with the same letters are not significantly different within a breeding method at P=5%.
