# Supplementary material for: Mate selection: A useful approach to maximize genetic gain and control inbreeding in genomic and conventional oil palm (Elaeis guineensis Jacq.) hybrid breeding
Source: PLoS Comput Biol. 2023 Sep 11;19(9):e1010290. doi: 10.1371/journal.pcbi.1010290 (PMC10513302; doi:10.1371/journal.pcbi.1010290)
Supplement: S6 Fig — Boxplots show distribution of values over 30 replicates. (DOCX) [file pcbi.1010290.s006.docx]

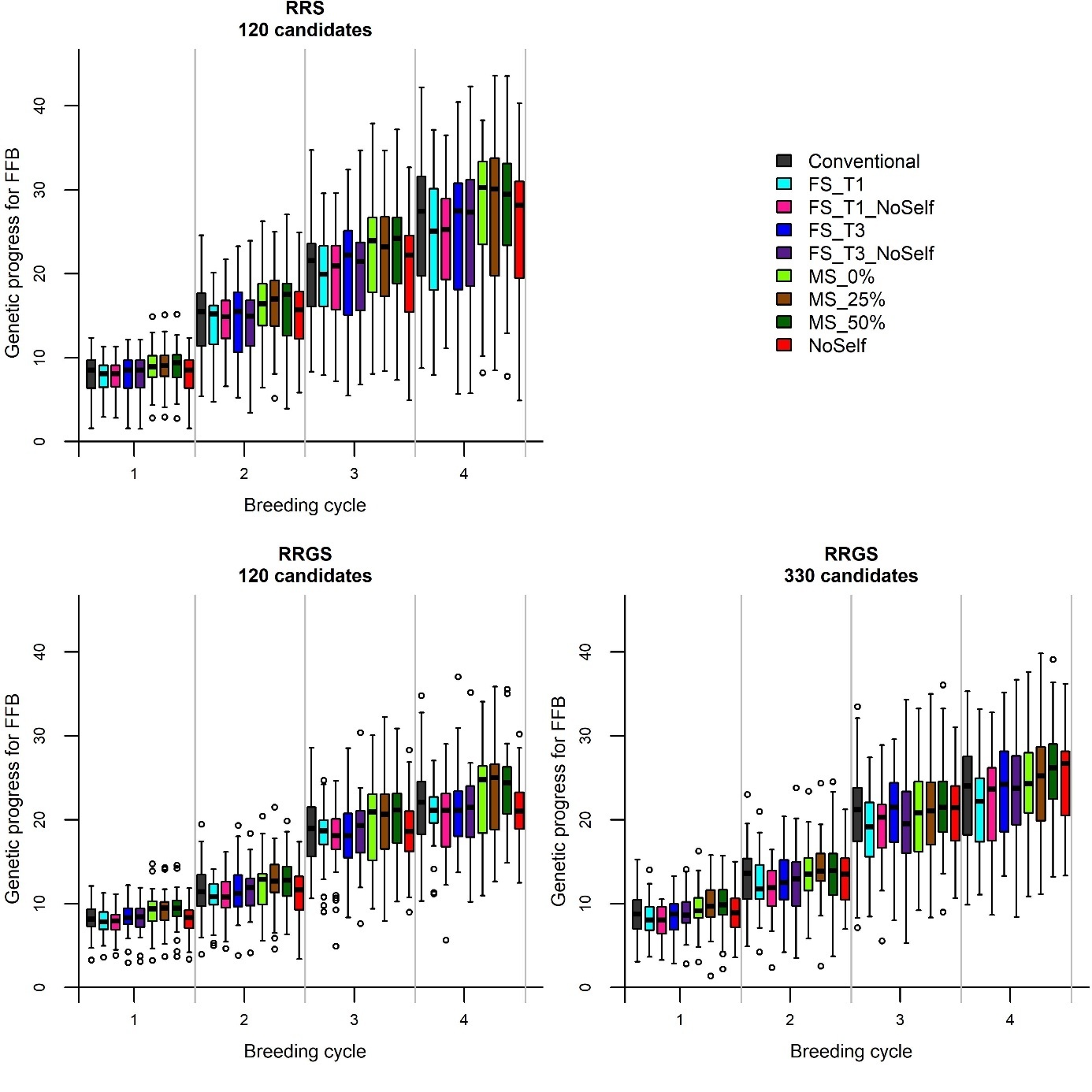


S6 Fig Genetic progress for FFB (in percentage of hybrids performance at generation 0) in the La Mé population according to breeding methods (RRS with 120 candidates, RRGS with 120 candidates and RRGS with 330 candidates), years (19 to 76 in RRS and 19 to 50 in RRGS) and methods of selection and mating. Boxplots show distribution of values over 30 replicates.
