# Supplementary material for: Mate selection: A useful approach to maximize genetic gain and control inbreeding in genomic and conventional oil palm (Elaeis guineensis Jacq.) hybrid breeding
Source: PLoS Comput Biol. 2023 Sep 11;19(9):e1010290. doi: 10.1371/journal.pcbi.1010290 (PMC10513302; doi:10.1371/journal.pcbi.1010290)
Supplement: S8 Fig — (DOCX) [file pcbi.1010290.s008.docx]

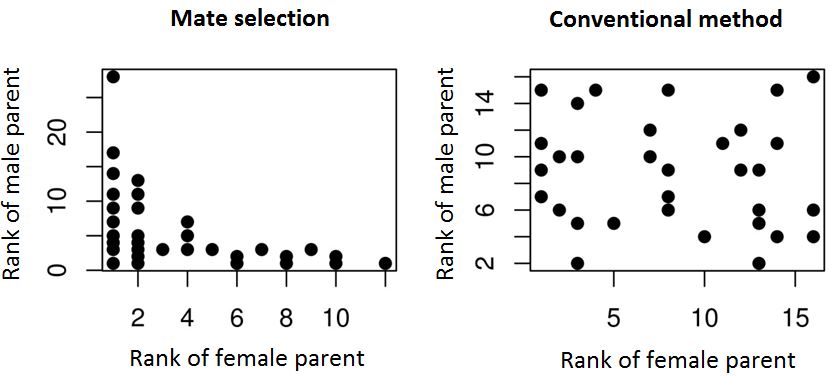


S8 Fig Example result showing the rank of the parent used as male and the rank of the parent used as female for the 32 crosses among the selected La Mé individuals, with mate selection and conventional selection
