# Supplementary material for: Mate selection: A useful approach to maximize genetic gain and control inbreeding in genomic and conventional oil palm (Elaeis guineensis Jacq.) hybrid breeding
Source: PLoS Comput Biol. 2023 Sep 11;19(9):e1010290. doi: 10.1371/journal.pcbi.1010290 (PMC10513302; doi:10.1371/journal.pcbi.1010290)
Supplement: Attachment — Submitted filename: Answers to reviewers comments_dc.docx [file pcbi.1010290.s009.docx]

Dear Editor,

Thanks for your efforts regarding our manuscript PCOMPBIOL-D-22-00885.

You will find in the attached file the detailed answers we made to the two reviewers. The changes we made appear in blue in the revised version of the manuscript. We believe all these comments largely improved the manuscript.

We hope you will find this new version suitable for publication in PLOS Computational Biology.

We remain at your disposal for any further details you might want.

Best regards,

David Cros

**Responses to the reviewers comments**

**Reviewer #1: Comments to authors**
In general, the paper is well written, and most contents are easy to follow. The paper investigated approaches of mate selection using computer simulations. Multiple breeding scenarios were tested, including a scenario with mate selection using simulated annealing optimization algorithms (SA), and some other conventional approaches. The paper found the mate selection using SA resulted in higher genetic gain and less increment of inbreeding across cycles, and provided reasonable discussions.

1. There is a main issue of the paper that needed to concern. In the paper, genetic gain of the target trait was tested in hybrid population. However, traits in this study were only simulated with additive genetic architecture. So, the paper is missing the power of capturing heterosis in hybrid population. Readers may be more interested in results from a simulation that both additive and dominance effects are simulated for traits (heterosis is from over-dominance).

Our article actually dealt with a particular aspect that, to our knowledge, was not addressed so far by the literature. When it comes to heterosis, multiplicative traits, i.e. those resulting from the product of basic components negatively correlated, represent a special case. Heterosis in multiplicative traits can be explained by a model without dominance, by the product of purely additive and complementary components (Gallais, 2009, p. 68-71; Schnell and Cockerham, 1992). In plants, multiplicative traits can include production, which is the product of the number of fruits multiplied by their average weight, and growth, which is the product of the number of nodes multiplied by the length of the internodes. Oil palm is thus a good model to study this type of traits, as total bunch production (FFB) is the product of the bunch number (BN) and the average bunch weight (BW), two mostly additive traits with a strong negative correlation (Gascon et al 1966, Corley et Tinker 2016).

In addition, the oil palm breeding populations are structured according to this correlation, with the Deli population having high BW and low BN, while the La Mé has low BW and high BN. This resulted in the implementation of reciprocal recurrent selection. In this context, the control of inbreeding is important in the parental populations as it might be detrimental for reproductive traits that affect seed production (e.g. seed germination). An extension of the current study would be to model reproductive traits in addition to BW and BN, and to simulate dominance effects for these traits (this is now mentioned in the discussion, see below).

Initially, however, we probably didn't emphasize the originality of our article enough. This is now explained in the introduction (lines 103-105, and lines 116-118) and in the discussion (see from line 665). This comes in addition to the explanation given in the first paragraph of “Simulation overview”.

2. Please at least add a paragraph in the Discussions to give details that recognizing the lack of dominance in the study, and the potential influences on the results, etc.

We added a paragraph in the discussion about this aspect, which is indeed important for the readers (see from line 665). This comes in addition to the explanation given in the first paragraph of “Simulation overview”.

3. In addition, the paper did not give info that whether the simulated annealing optimization algorithm was coded in-house or from published package. Please add some info in Methods. and if coded in-house, would be great to make it public for people to re-use in practise.

We used custom R scripts that we developed specifically for the study. This is now mentioned in “Simulation overview” and in the “Data availability” sections.

Overall, the manuscript needs intermediate level of revision, especially on Methods and Results. Details as below.

***P5 line 18, marker-assisted selection (MAS) is a term for a specific approach that applied couple decades ago, and in theory, genomic selection is different to MAS. Please remove marker-assisted selection to avoid misleading. The comment applies across the manuscript.***

Done.

***P10 line 124, remove ‘indeed’***

Done.

***P11 line 143, please state what items were compared between scenarios, e.g. genetic gain of target trait and inbreeding level between breeding scenarios.***

Done.

***P12 line 167, ‘R-ASReml’ change to ‘ASReml-R’***

Done.

**P12 line 187-190, please give more detail on how you sampled QTL effects, e.g. effects sampled from normal distribution? QTL positions sampled randomly from SNPs?
And, you only mentioned the traits were simulated following additive genetic architecture (line 157). Please add more detail of how phenotypes simulated, e.g. genetic variance + error term, and the error term evaluated from heritability, etc.
In addition, it is better to show the genetic correlation between the traits after ‘pleiotropic QTLs’ (line 189), rather than putting those info in Suppl. Table.**

Done.

**P13 line 196, for readers’ conveniences and making the paper self-contained, please add a figure to make breeding schemes visualized in the paper, rather than simply referring to other papers.**

Done (see new Figure 1).

**P15 line 257, ‘matrix X’ changes to ‘matrix X’ (‘X’ in bold). Please revise any matrix notations to bold across the manuscript.**

Done.

**P15 line 263, Let’s change to ‘Let’**

Done.

**P15 line 264, please revise notations, e.g. ‘DiDj’ should be ‘DiDj’ (subscript for ‘i’ and ‘j’). This comment applies to others across the manuscript (e.g. LMiLMj, etc.)**

Done.

**P16 line 276, ‘five first iterations’ changes to ‘first five iterations’**.

Done.

**P18 line 317 - 319, the contents repeated in the table, can be removed from the manuscript.**

Done.

**P19 line 342, ‘calcInbreeding’ is not a default function in R, please give details of package.**

Done.

**P19 line 358-359. Suppl. Figure 3 came before Suppl. Figure 2 in the text of manuscript, please swap.**
Done.

**In addition, the colours used in all figures are hard to be distinguished, especially in line charts, e.g. Fig 1 ‘RRGS 330 candidates’. Please re-plot all figures with colours easy to tell apart.**

We used a new set of colors easier to distinguish.

**P20 line 390, the sentence can be moved to line 389 as one paragraph.**

Done.

**P21 line 414-476, the whole section of ‘selection and mating’ in Results is lack of info in Methods, which making it hard to follow. Please add some info in Methods, e.g. what are the items and how they were analysed, etc.
In addition, some contents in this section should belong to Discussions, e.g. P23 line 452.
Please revise the whole section, and relocate some contents to Methods / Discussions.**

We added and extra paragraph at the end of the material and method to explain the different parameters considered to understand the solutions retained by the simulated annealing process with mate selection.

Also, some elements of the ‘selection and mating’ were moved to the discussion.

**P25 line 500-519, the paragraph could be moved to the end of Discussions**

This was move towards the end of discussion.

**P25 line 507 -509, the info is vague, better to give details of how long the convergence time if optimizing both the La Me and Deli population against the current one optimizing La Me only.**

We gave additional information (although unfortunately we did not keep records of detailed computation times).

**P28 line 579 – 596, the paragraph could be removed. The core of the study is comparing mate selection with other conventional approaches. However, this paragraph is about different ways of measuring inbreeding, and non-related to your objectives.**

We initially included this paragraph as we considered that readers might wonder why we did not use ROH to measure inbreeding, while this approach gained a lot of popularity. As the reviewer 2 found this paragraph useful we prefer keeping it, although in a shorter version to take into consideration the present comment (and we integrated the comment from reviewer 2).

**P28 line 597 – 599, can be moved to the end of line 596 (or removed).**

Done.

**Please add two paragraphs at the end of Discussions, one regarding the simulation of dominance (as mentioned before), and one to formally conclude the paper (conclusion, ideas for further studies, etc.)**
The two paragraphs were added (see from line 675 and from line 691).

**Suppl Table 1. The contents are important for simulations. Strongly recommend to put it in the main manuscript, rather than in supplementary.**

Done.

**Reviewer #2:** Mate selection (MS) program have been studied extensively especially in animal breeding programs, so the concept itself is not new. This is nicely written paper addressing the important question of balancing inbreeding and genetic gain in the long-term conventional breeding and molecular breeding schemes of oil palm. There are, however, some limitations of this study as indicated in my comments below. I have several questions and suggestions for the authors to consider:

**1. I suggest including a diagram showing the simulated scheme as the description is somewhat confusing for readers who are not familiar with this crop.**

Done (see new Figure 1).

**2. MS scheme was shown to result in higher inbreeding and higher genetic gain (Figures 2 and 3), for a largely additive genetic trait, over the longer term. It is important to note that with a given inbreeding level, the deleterious load could be different for different traits – hence the genetic gain will vary. Therefore, it is not realistic to generalize the utility of MS scheme for economic traits that are influenced by non-additive gene action. The question is how to design a MS scheme for a breeding program where some of the key traits are controlled by different gene actions (e.g., additive and non-additive).**

Please see our answer to questions 1. and 2. Of reviewer 1, who raised the same point.

**3. The interpretations of results in this study are mainly focused on comparing different mating and selection schemes. I would suggest authors to also highlight and compare the results between RRS and RRGS schemes.**

# This is a good idea as the article also shows the superiority of RRGS over RRS. We initially did not emphasize this aspect as it already appeared in our previous simulation articles (Cros et al 2015 BMC genomics, Cros et al 2018, Molecular Breeding). However, based on the reviewer comment, we now briefly compare the two breeding schemes throughout the document (see, in results, the end of “Inbreeding measured from pedigree and SNP” and “Genetic progress”), and we added a paragraph about that in the discussion (see from line 656 to 674).

**4. Empirical data from commercial species suggest that inbreeding per generation (and per year) can increase faster with GS schemes, but apparently SNP-based inbreeding in this study was higher for RRS scheme compared with RRGS for a given size of selection population (N=120; Figures 1 and 2). Please clarify/discuss these results.**

Based on this comment, we now explain this aspect in results (see from line 424) and we discuss it (see from line 665).

**5. Figures 3 and 4: For the same size of selection candidates (N=120), why the genetic progress after 4 cycles of breeding is higher for RRS scheme compared to RRGS, although the gain per year is obviously higher for the RRGS schemes. Does it mean that RRGS is less efficient/accurate than RRS for the traits investigated in this study?**

The lower genetic progress after 4 cycles with RRGS (Figure 4) comes from the fact that in oil palm conventional selection based on progeny tests has a greater prediction accuracy of genetic values (r ~ 0.8-0.9, see Cros et al 2015 TAAG) compared to GS. This was noted in our previous simulation studies (see Cros et al 2015 BMC Genomics), as well as in empirical studies (see Cros et al 2017). This is likely due to the limited size of the training populations available so far in this species. However, the decrease in number of years for 4 cycles of RRS (76 years) against 4 cycles of RRGS (50) is stronger than the reduction in prediction accuracy, and therefore the annual genetic progress is higher with RRGS than with RRS (Figure 5). This is in agreement with previous results (Cros et al 2015 BMC Genomics, Cros et al 2018 Mol breeding). This is now explained at the end of the discussion.

**6. Figures 1 and 2: For the two RRGS schemes, why is inbreeding higher for the larger selection population of 330 compared to 120 candidates? Unless I have missed something, I find these results counterintuitive. Please clarify.**

This came from the fact that the number of crosses within a parental population to generate the next generation is fixed (32, see material and method). In these conditions, generating a larger population (330 instead of 120) leads to larger families of full-sibs, which gives the possibility of selecting more full-sibs and thus to reach higher levels of inbreeding. This is now explained in the results section (see line 420).

The fixed number of crosses was chosen for two reasons: (i) to match with the practices of oil palm breeders, considering that making more crosses could be difficult, and (ii) for computational reasons, as this would increase the space of possibilities for the optimization algorithm behind mate selection. However, varying this parameter would be of interest for future studies, and this is mention in the discussion part (line 647).

**7. Figures 3 and 4: Genetic progress is higher for the increased levels of inbreeding. Perhaps it shows that the trait investigated in this study is not affected by inbreeding depression – hence not a good trait to find a balance between inbreeding and genetic gain, which is perhaps one of the main focuses of this study. A simulated inbreeding management strategy would perhaps allow discrimination between 2 individuals, with same genomic inbreeding coefficient, based on the amount of deleterious load each carry. Please provide your thoughts on the choice of trait in this context.**

We think the article did not emphasize enough the rationale behind the choice of the trait of interest (FFB) and the additive modeling of the two base traits generating the FFB (BN and BW). Please see our answer to the question 1 of reviewer 1,

**8. Line 593-595: Genealogical inbreeding levels were very low compared with those computed over SNPs, but perhaps the realized values could be somewhere in-between as the latter are upwardly biased. I guess it might be very useful to present ROH-based genomic inbreeding level even if these are highly correlated with SNP-level inbreeding values – just to check the magnitude of inbreeding.**

The realized values indeed seem to be between the inbreeding given by the pedigree and single SNP-based analyses. This is now mentioned in the discussion (line 621). For example, in generation 0, using the consecutiveRUNS function of the detectRuns R package and considering ROH with at least 5 SNP, the ROH-based genomic inbreeding was 0.44 on average over the different replicates, against 0.13 for pedigree-based inbreeding and 0.73 for SNP-based inbreeding. The ROH approach is highly informative, as suggested by the reviewer, and we plan to apply it in a future study to investigate the history of our populations using real data.

**9. Figures 6 and 7: For the MS scheme, the maximum rank of selected individuals, and the number of crosses per selected individual, was not affected by the inbreeding constraint () when the size of selection population was 120 (as opposed to 330). Why only the larger selection population size is impacting this variable?**

This is linked to the fact that the number of crosses has been fixed (see answer to question 6). Under these conditions, generating a larger population (330 instead of 120) leads to larger families of full-sibs. This increases the need for inbreeding management and therefore the possibility of having the MS optimization algorithm adopt contrasting behaviors for different levels of cΔF. For example, this means that it is more likely to select lower-ranked individuals to represent a greater number of full-sib families with 330 candidates than with 120. This should be clearer now with lines 464-466.

**Does your statement (Line 452-254) “mate selection adjusted the number of crosses per individual to optimize the genetic gain rather than to limit inbreeding” suggest that MS is not the right tool to balance inbreeding and genetic gain in the long-term for this breeding program?**

This comment was about the parameter “correlation between rank and number of crosses” (“*correlation between rank and number of crosses was not affected by cΔF, suggesting that mate selection adjusted the number of crosses per individual to optimize the genetic gain rather than to limit inbreeding*").

MS can balance inbreeding and genetic gain in the long-term for this breeding program (giving more or less weight to inbreeding and genetic gain depending on cΔF), as mentioned for example at the beginning of the discussion and at the beginning of the final paragraph that was added based on the reviewer comments. However it seems that, to achieve this balance, the optimization algorithm does not rely on a variation in the number of times each individual is mated that would be correlated with its rank. It therefore mobilizes other parameters, such as the percentage of selfings (more selfings are allowed when the inbreeding constraint is relaxed, leading to more inbreeding but higher genetic progress).

**10. Supplementary Table 1: Does the value for additive variance represent interpopulation or intrapopulation variance? Please clarify.**

This is the value for interpopulation additive variance, ie which expresses in hybrid crosses, as indicated in the caption of the table and in the table. To clarify this, we added the reference giving the formula we used to compute this variance.

**11. I will encourage authors to draw on the learnings from applications of mate selection schemes in animal breeding programs to interpret the results from this study. The patterns of changes in inbreeding level and genetic gain would be somewhat comparable between the plant breeding and animal breeding schemes.**

Thanks to this comment, we improved the discussion and included 4 new references taken from the animal breeding community, and in particular aquaculture breeding (see lines 580-590, and 602-603).
